# Low- and middle-income countries demonstrate rapid growth of type 2 diabetes: an analysis based on Global Burden of Disease 1990–2019 data

**DOI:** 10.1007/s00125-022-05713-6

**Published:** 2022-05-19

**Authors:** Jinli Liu, Ruhai Bai, Zhonglin Chai, Mark E. Cooper, Paul Z. Zimmet, Lei Zhang

**Affiliations:** 1 China-Australia Joint Research Center for Infectious Diseases, School of Public Health, Xi’an Jiaotong University Health Science Center, Xi’an, Shaanxi China; 2 Department of Epidemiology and Biostatistics, School of Public Health, Peking University, Beijing, China; 3 School of Public Affairs, Nanjing University of Science and Technology, Nanjing, China; 4 Department of Diabetes, Central Clinical School, Monash University, Melbourne, VIC Australia; 5 Melbourne Sexual Health Centre, Alfred Health, Melbourne, VIC Australia; 6 Central Clinical School, Faculty of Medicine, Monash University, Melbourne, VIC Australia; 7 Department of Epidemiology and Biostatistics, College of Public Health, Zhengzhou University, Zhengzhou, Henan China

**Keywords:** DALYs, Death, Population attributable fraction, Type 2 diabetes mellitus

## Abstract

**Aims/hypothesis:**

The study aims to quantify the global trend of the disease burden of type 2 diabetes caused by various risks factors by country income tiers.

**Methods:**

Data on type 2 diabetes, including mortality and disability-adjusted life years (DALYs) during 1990–2019, were obtained from the Global Burden of Disease Study 2019. We analysed mortality and DALY rates and the population attributable fraction (PAF) in various risk factors of type 2 diabetes by country income tiers.

**Results:**

Globally, the age-standardised death rate (ASDR) attributable to type 2 diabetes increased from 16.7 (15.7, 17.5)/100,000 person-years in 1990 to 18.5 (17.2, 19.7)/100,000 person-years in 2019. Similarly, age-standardised DALY rates increased from 628.3 (537.2, 730.9)/100,000 person-years to 801.5 (670.6, 954.4)/100,000 person-years during 1990–2019. Lower-middle-income countries reported the largest increase in the average annual growth of ASDR (1.3%) and an age-standardised DALY rate (1.6%) of type 2 diabetes. The key PAF attributing to type 2 diabetes deaths/DALYs was high BMI in countries of all income tiers. With the exception of BMI, while in low- and lower-middle-income countries, risk factors attributable to type 2 diabetes-related deaths and DALYs are mostly environment-related, the risk factors in high-income countries are mostly lifestyle-related.

**Conclusions/interpretation:**

Type 2 diabetes disease burden increased globally, but low- and middle-income countries showed the highest growth rate. A high BMI level remained the key contributing factor in all income tiers, but environmental and lifestyle-related factors contributed differently across income tiers.

**Data availability:**

To download the data used in these analyses, please visit the Global Health Data Exchange at http://ghdx.healthdata.org/gbd-2019.

**Graphical abstract:**

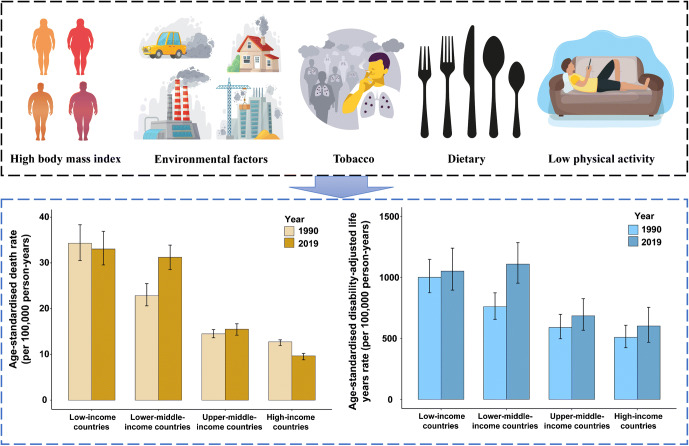

**Supplementary Information:**

The online version of this article (10.1007/s00125-022-05713-6) contains peer-reviewed but unedited supplementary material.

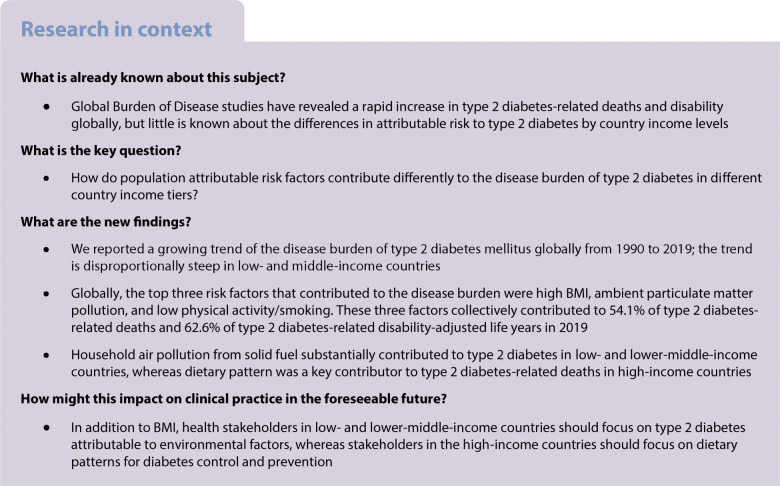



## Introduction

Diabetes mellitus is a fast-growing ongoing global health emergency of the twenty-first century [1, 2]. Studies show that diabetes alone accounted for 3.96 million deaths worldwide in 2010 [3], and this number increased to 4.20 million among adults in 2019 [1]. Diabetes has emerged as the fourth leading cause of disability globally, and the number of disability-adjusted life years (DALYs) caused by diabetes was 66.3 million globally in 2019 [4]. Type 2 diabetes is the most common type of diabetes and accounts for around 90% of all diabetic cases worldwide [1, 2, 5]. The economic development and improvement in healthcare in individual countries have a considerable impact on the disease burden of type 2 diabetes. In many developed countries, including Scotland [6], Canada [7], UK [8], Denmark [9], Sweden [10], Australia [11] and USA [12], type 2 diabetes-related mortality has declined steadily. People living with type 2 diabetes are at higher risk of chronic diseases, viral hepatitis and even COVID-19 severity [13–16]. However, large developing economies such as China reported an ongoing and increasing trend of diabetes mellitus, with diabetes mortality increasing from 5.3 deaths/100,000 people in 1990 to 10.9 deaths/100,000 people in 2017 [17]. In developing countries with limited resources, inaccessibility to type 2 diabetes medications and other treatments [18] is an important cause of type 2 diabetes-related mortality and disability. Many resource-limited countries employed intensive blood glucose control, which unexpectedly increased the risk of hypoglycaemia and potential death in its population [19]. An epidemiological study suggested that during 2010–2030, the number of adults with diabetes mellitus in developing countries would increase by 69%, which is more than threefold the 20% predicted increase in developed countries [20].

The Global Burden of Disease (GBD) study indicated that high BMI was the leading risk factor contributing to 41.2% type 2 diabetes mortality and 54.0% DALYs, followed by ambient particulate matter pollution risk factors (accounting for 18.0% and 18.3% of type 2 diabetes-related deaths and DALYs), and diet low in whole grains (accounting for 16.5% and 21.2% of type 2 diabetes-related deaths and DALYs) in 2017 [21] followed. However, these previous studies did not address the impact of diabetes on mortality and disability attributable to major risk factors in countries at different income levels. Understanding this knowledge will enable policymakers to tailor their policies to adjust for the various levels of economic development in these countries. This will provide informed strategies for coordinated actions to improve their health and social security systems to mitigate diabetes-related mortality and disability. In this study, we aimed to estimate type 2 diabetes-related deaths and DALYs attributable to various modifiable risk factors over the period of 1990–2019 in countries at various income tiers.

## Methods

### Overview

The GBD 2019 estimation of attributable burden followed the general framework established for comparative risk assessment [22, 23] used in GBD since 2002 [24]. GBD 2019 estimated prevalence of exposure and attributable deaths, years of life lost (YLLs), years lived with disability (YLDs), and DALYs for 23 age groups; men, women, and both sexes combined; 87 behavioural, environmental and occupational, and metabolic risk factors; reported estimates for 369 diseases and injuries; and 203 countries (Taiwan: province of China) and territories that were grouped into 21 regions and seven super-regions [4, 25]. All available data on causes of death are standardised and pooled into a single database used to generate cause-specific mortality estimates by age, sex, year and geography [4]. In GBD 2019, DALYs were computed by adding YLLs and YLDs for each cause, location, age group, sex and year.

The attributable burden is the potential reduction in the current disease burden if past population exposure had shifted to an alternative or counterfactual distribution of risk exposure. In this study, we included 13 risk factors introduced in the GBD studies. These attributable risk factors include metabolic (i.e. high BMI), environmental and occupational (i.e. ambient particulate matter pollution and household air pollution from solid fuels), behavioural (i.e. tobacco [smoking and second-hand smoking], dietary [diet low in whole grains, diet low in fruits, diet low in nuts and seeds, diet high in sugar-sweetened beverages, diet high in red meat, diet low in fibre and diet high in processed meat], and low physical activity). The definition of these risk factors has been provided in detail in previous publications [26]. The GBD 2019 study adopted a set of unified RRs for all attribution analysis globally [25]. In brief, the RRs were modelled using meta-regression with pooled data from prospective cohort studies or published literature reviews. For each risk–outcome pair, we used the expected summary exposure value (SEV) to calculate the expected population attributable fractions (PAFs) [4, 25]. The method of evaluating the impact of environmental pollutants has been described in a GBD study [25].

Socioeconomic status was defined based on the gross national per capita income, as classified by the World Bank, i.e. low-income countries (LICs), lower-middle-income countries (LMICs), upper-middle-income countries (UMICs), and high-income countries (HICs) [27].

### Disease burden attributable to risk factors

For continuous risk factors, such as BMI, the formula for the PAF is defined as:

$$ \mathrm{PAF}=\frac{\int_{x=0}^m RR(x){P}_1(x) dx-{\int}_{x=0}^m RR(x){P}_2(x) dx}{\int_{x=0}^m RR(x){P}_1(x) dx} $$

Where *RR*(*x*) is the *RR* of a certain disease for exposure level *x*, *P*_1_(*x*) is the population distribution of the exposure, *P*_2_(*x*) is the minimum theoretical exposure distribution, and *m* is the maximum exposure level. The minimum theoretical exposure is the counterfactual condition of exposure and has been previously defined for each risk factor [26]. For categorical risk factors (e.g. smoking and physical inactivity), the formula for PAF is:

$$ \mathrm{PAF}=\frac{\sum \limits_{i=1}^n{P}_i\left({RR}_i-1\right)}{\sum \limits_{i=1}^n{P}_i\left({RR}_i-1\right)+1} $$

where *i* is exposure level, *RR*_*i*_ is *RR* for exposure level *i*, and *P*_*i*_ is the prevalence of exposure level *i*.

For calculating the burden of multiple risk factors, validation studies have reported congruency between the true risk associated with multiple risk factors affecting the same outcome and a multiplicative aggregation of the PAFs of the individual risk factors [28].

We calculated the joint PAF of multiple risk factors based on the following formula. The mediation factors were estimated using GBD 2019 [25].

$$ {PAF}_{oast}=1-{\prod}_{j=1}^J\left[1-{PAF}_{ioast}{\prod}_{j=1}^J\left(1-{MF}_{jio}\right)\right] $$

where *J* is the number of risk factors for calculating the joint effect, *PAF*_*ioast*_ is the attributable fraction of *i* risk factor, *MF*_*jio*_ is the mediation factor between risk factor *i* and a certain disease *o* through risk factor *j*, *a* was the age group, *s* was sex and *t* was year.

### Indicators of annual change in the burden of disease

The rate of annual growth was measured as the average annual change in type 2 diabetes burden from 1990 to 2019:

$$ \mathrm{Annual}\ \mathrm{growth}=\frac{\left(\mathrm{Value}\ \mathrm{in}\ 2019-\mathrm{Value}\ \mathrm{in}\ 1990\right)}{\mathrm{Value}\ \mathrm{in}\ 1990\times 29} $$

All statistical analyses were performed using the R v3.5.1 (https://www.r-project.org/). A probability value of *p*<0.05 was considered statistically significant.

## Results

### Global type 2 diabetes over time

Globally, the number of deaths attributable to type 2 diabetes increased from 0.61 (95% CI 0.57, 0.64) million in 1990 to 1.47 (1.37, 1.57) million in 2019, with an annual growth rate of 4.9% (4.6, 5.2). The age-standardised risk-attributable death rates increased from 16.7 (15.7, 17.5)/100,000 person-years to 18.5 (17.2, 19.7)/100,000 person-years over the same period (0.4% [0.1, 0.7] annual growth, Table 1). Similarly, the number of DALYs attributable to type 2 diabetes increased from 25.48 (21.70, 29.78) million in 1990 to 66.30 (55.48, 79.01) million in 2019 (annual growth 5.5% [4.7, 6.3]), corresponding to an increase of age-standardised DALY rates from 628.3 (537.2, 730.9)/100,000 person-years to 801.5 (670.6, 954.4)/100,000 person-years (annual growth 1.0% [0.2, 1.7]) during 1990–2019 (Table 1).

**Table 1:** Type 2 diabetes-related mortality and DALYs indicators by country income tiers, 1990–2019

Variable	Mortality cases (×10^3^, 95% CI)			ASDR (/100,000, 95% CI)			DALYs (No. ×10^3^, 95% CI)			Age-standardised DALY rate (/100,000, 95% CI)		
	1990	2019	Annual growth (%)	1990	2019	Annual growth (%)	1990	2019	Annual growth (%)	1990	2019	Annual growth (%)
Global												
All factors	606 (573, 638)	1473 (1372, 1566)	4.9 (4.6, 5.2)	16.7 (15.7, 17.5)	18.5 (17.2, 19.7)	0.4 (0.1, 0.7)	25,478 (21,701, 29,776)	66,300 (55,477, 79,005)	5.5 (4.7, 6.3)	628.3 (537.2, 730.9)	801.5 (670.6, 954.4)	1.0 (0.2, 1.7)
Metabolic factors												
High BMI	191 (118, 276)	619 (436, 815)	7.7 (7.1, 8.3)	5.0 (3.0, 7.3)	7.6 (5.3, 10.0)	1.8 (0.4, 3.2)	9391 (5778, 13,913)	34,422 (24,110, 46,308)	9.2 (8.7, 9.7)	224.8 (137.9, 332.0)	411.1 (287.5, 552.2)	2.9 (1.6, 4.1)
Environmental factors												
Ambient particulate matter pollution	56 (37, 79)	197 (136, 258)	8.7 (7.8, 9.6)	1.6 (1.0, 2.2)	2.5 (1.7, 3.2)	2.0 (0.6, 3.4)	2330 (1463, 3318)	9034 (6135, 12,213)	9.9 (9.1, 10.7)	58.4 (36.6, 83.1)	109.0 (74.1, 147.2)	3.0 (1.6, 4.4)
Household air pollution from solid fuels	75 (49, 121)	96 (61, 138)	1.0 (−0.9, 2.9)	2.0 (1.3, 3.2)	1.2 (0.7, 1.7)	−1.4 (−3.7, 0.9)	3126 (1984, 5001)	3922 (2432, 5851)	0.9 (−1.1, 2.9)	75.9 (48.1, 120.4)	47.1 (29.2, 70.4)	−1.3 (−3.6, 1.0)
Tobacco												
Smoking	67 (56, 80)	121 (99, 145)	2.7 (1.9, 3.6)	1.8 (1.5, 2.1)	1.5 (1.2, 1.8)	−0.5 (−1.5, 0.4)	3278 (2559, 4071)	6541 (4997, 8273)	3.4 (2.3, 4.5)	79.2 (61.9, 97.9)	78.1 (59.6, 98.7)	−0.1 (−1.2, 1.1)
Second-hand smoke	56 (22, 84)	123 (48, 189)	4.2 (1.7, 6.7)	1.5 (0.6, 2.3)	1.5 (0.6, 2.4)	0.1 (−2.8, 3.0)	2462 (925, 3868)	5803 (2138, 9191)	4.7 (2.1, 7.3)	60.0 (22.5, 94.1)	69.7 (25.7, 110.5)	0.6 (−2.4, 3.5)
Dietary												
Diet low in fruits	41 (26, 57)	88 (56, 126)	4.0 (2.4, 5.7)	1.1 (0.7, 1.5)	1.1 (0.7, 1.6)	0 (−1.9, 1.9)	1784 (1112, 2574)	3946 (2370, 5908)	4.2 (2.3, 6.1)	43.6 (27.0, 62.9)	47.6 (28.6, 71.3)	0.3 (−1.7, 2.3)
Diet low in whole grains	31 (11, 46)	73 (26, 108)	4.7 (2.1, 7.2)	0.9 (0.3, 1.3)	0.9 (0.3, 1.4)	0.2 (−2.7, 3.2)	1289 (451, 1975)	3300 (1129, 5144)	5.4 (2.7, 8.1)	32.0 (11.2, 49.0)	39.9 (13.7, 62.1)	0.8 (−2.1, 3.8)
Diet high in red meat	35 (19, 48)	82 (46, 115)	4.7 (3.0, 6.5)	1.0 (0.5, 1.3)	1.0 (0.6, 1.5)	0.2 (−1.8, 2.3)	1524 (873, 2207)	4123 (2434, 5962)	5.9 (4.2, 7.5)	37.9 (21.8, 54.6)	49.8 (29.4, 72.0)	1.1 (−0.9, 3.0)
Diet high in processed meat	34 (24, 41)	73 (51, 88)	3.9 (2.8, 5.1)	1.0 (0.7, 1.2)	0.9 (0.6, 1.1)	−0.2 (−1.5, 1.1)	1447 (996, 1891)	3680 (2431, 4883)	5.3 (3.8, 6.8)	36.5 (25.2, 47.6)	44.6 (29.5, 59.0)	0.8 (−0.8, 2.3)
Diet high in sugar-sweetened beverages	20 (14, 26)	49 (30, 64)	4.8 (3.1, 6.5)	0.6 (0.4, 0.7)	0.6 (0.4, 0.8)	0.3 (−1.3, 1.9)	864 (578, 1140)	2334 (1385, 3239)	5.9 (3.9, 7.9)	21.4 (14.3, 28.2)	28.2 (16.8, 39.1)	1.1 (−0.7, 2.9)
Diet low in fibre	20 (10, 31)	41 (17, 65)	3.5 (0.9, 6.0)	0.6 (0.3, 0.9)	0.5 (0.2, 0.8)	−0.3 (−3.0, 2.4)	843 (380, 1318)	1779 (727, 2876)	3.8 (1.2, 6.5)	20.9 (9.5, 32.7)	21.6 (8.8, 34.9)	0.1 (−2.7, 2.9)
Diet low in nuts and seeds	13 (3, 26)	29 (8, 55)	4.2 (1.3, 7.2)	0.4 (0.1, 0.7)	0.4 (0.1, 0.7)	0 (−3.7, 3.7)	551 (129, 1105)	1316 (372, 2568)	4.8 (1.8, 7.7)	13.7 (3.2, 27.4)	15.9 (4.5, 31.1)	0.6 (−3.0, 4.2)
Physical activity												
Low physical activity	50 (25, 85)	125 (62, 208)	5.2 (3.0, 7.4)	1.5 (0.8, 2.5)	1.6 (0.8, 2.7)	0.3 (−2.2, 2.9)	1720 (782, 3071)	4549 (2189, 7969)	5.7 (3.5, 7.9)	45.0 (21.3, 79.5)	55.9 (27.2, 97.6)	0.8 (−1.8, 3.5)
LICs												
All factors	44 (39, 50)	88 (78, 99)	3.4 (2.9, 3.9)	34.3 (30.5, 38.3)	33.0 (29.6, 36.9)	−0.1 (−0.7, 0.4)	1556 (1348, 1797)	3471 (2924, 4100)	4.2 (3.5, 5.0)	999.6 (873.7, 1147.3)	1050.9 (895.7, 1238.9)	0.2 (−0.5, 0.9)
Metabolic factors												
High BMI	9 (4, 16)	30 (18, 43)	7.5 (6.9, 8.2)	6.2 (2.7, 10.9)	9.6 (5.7, 14.2)	1.9 (−0.1, 3.9)	387 (182, 656)	1438 (903, 2047)	9.4 (9.2, 9.6)	226.0 (105.4, 382.8)	393.7 (245.0, 566.0)	2.6 (0.9, 4.3)
Environmental factors												
Ambient particulate matter pollution	1 (0, 2)	4 (2, 7)	12.4 (11.9, 12.9)	0.7 (0.2, 1.5)	1.5 (0.7, 2.6)	4.2 (2.1, 6.4)	31 (10, 69)	162 (78, 280)	14.6 (14.6, 14.6)	20.2 (6.3, 45.1)	50.0 (24.0, 85.5)	5.1 (3.1, 7.0)
Household air pollution from solid fuels	12 (7, 24)	19 (12, 30)	2.0 (−0.1, 4.0)	9.4 (5.7, 18.5)	7.1 (4.7, 11.2)	−0.8 (−3.5, 1.9)	415 (248, 842)	722 (466, 1157)	2.6 (0.6, 4.5)	270.0 (161.9, 537.1)	221.8 (144.0, 352.6)	−0.6 (−3.3, 2.0)
Tobacco												
Smoking	3 (2, 3)	5 (3, 6)	2.5 (1.2, 3.7)	1.9 (1.3, 2.4)	1.5 (1.1, 2.0)	−0.6 (−2.0, 0.8)	98 (72, 125)	196 (143, 261)	3.5 (2.2, 4.7)	61.1 (44.6, 77.9)	57.6 (42.2, 76.2)	−0.2 (−1.6, 1.2)
Second-hand smoke	3 (1, 4)	5 (2, 8)	3.4 (0.8, 6.0)	2.0 (0.8, 3.2)	1.9 (0.7, 3.0)	−0.2 (−3.2, 2.8)	101 (38, 160)	230 (83, 369)	4.4 (1.7, 7.1)	64 (24.1, 101.5)	67.6 (24.6, 108.6)	0.2 (−2.8, 3.2)
Dietary												
Diet low in fruits	3 (2, 5)	7 (4, 9)	3.4 (1.8, 5.0)	2.6 (1.8, 3.6)	2.6 (1.7, 3.5)	−0.1 (−1.8, 1.6)	120 (78, 162)	265 (170, 371)	4.2 (2.6, 5.8)	77.2 (50.8, 104.0)	80.9 (52.6, 112.6)	0.2 (−1.5, 1.9)
Diet low in whole grains	2 (1, 3)	4 (1, 7)	3.3 (0.5, 6.1)	1.7 (0.6, 2.6)	1.6 (0.5, 2.5)	−0.2 (−3.4, 3.0)	77 (26, 119)	170 (55, 263)	4.1 (1.4, 6.9)	50.2 (16.7, 77.5)	51.9 (16.8, 79.9)	0.1 (−3.0, 3.3)
Diet high in red meat	1 (0, 2)	2 (1, 4)	3.7 (0.5, 6.8)	0.9 (0.2, 1.4)	0.9 (0.2, 1.4)	0 (−3.7, 3.7)	43 (11, 71)	99 (25, 169)	4.6 (1.5, 7.6)	26.8 (6.5, 44.8)	29.4 (7.3, 49.7)	0.3 (−3.2, 3.9)
Diet high in processed meat	2 (1, 2)	4 (2, 5)	4.0 (2.4, 5.7)	1.3 (0.7, 1.7)	1.4 (0.8, 1.8)	0.2 (−1.8, 2.1)	65 (36, 84)	159 (90, 211)	5.0 (3.4, 6.7)	40.5 (21.9, 52.7)	46.9 (26.5, 61.9)	0.5 (−1.4, 2.4)
Diet high in sugar-sweetened beverages	1 (1, 2)	2 (2, 3)	2.5 (1.3, 3.7)	1.0 (0.8, 1.2)	0.8 (0.6, 1.1)	−0.6 (−1.7, 0.6)	44 (34, 55)	85 (59, 111)	3.2 (1.8, 4.6)	28.9 (22.5, 35.8)	26.3 (19.0, 33.8)	−0.3 (−1.6, 0.9)
Diet low in fibre	1 (0, 1)	2 (1, 3)	3.3 (0.6, 6.0)	0.7 (0.3, 1.2)	0.6 (0.2, 1.1)	−0.3 (−3.5, 2.9)	31 (11, 52)	70 (27, 119)	4.4 (1.8, 6.9)	20.4 (7.2, 34.0)	21.2 (8.1, 35.6)	0.1 (−2.9, 3.2)
Diet low in nuts and seeds	1 (0, 2)	1 (0, 3)	2.4 (−1.2, 6.1)	0.6 (0.1, 1.2)	0.5 (0.1, 1.1)	−0.5 (−4.6, 3.5)	27 (7, 54)	53 (10, 111)	3.2 (−0.4, 6.9)	17.8 (4.4, 35.4)	16.3 (3.3, 34.3)	−0.3 (−4.2, 3.7)
Physical activity												
Low physical activity	2 (1, 4)	4 (2, 8)	4.7 (2.3, 7.1)	1.6 (0.7, 3.0)	1.8 (0.8, 3.2)	0.4 (−2.5, 3.3)	57 (24, 113)	147 (61, 289)	5.5 (2.9, 8.2)	40.9 (17.9, 79.8)	49.7 (21.8, 93.3)	0.7 (−2.2, 3.7)
LMICs												
All factors	201 (182, 224)	649 (596, 704)	7.7 (7.5, 7.9)	22.8 (20.6, 25.5)	31.2 (28.5, 33.9)	1.3 (0.9, 1.7)	8246 (7050, 9516)	27,137 (23,209, 31,659)	7.9 (7.3, 8.5)	760.0 (656.1, 873.0)	1108.3 (951.6, 1285.5)	1.6 (0.9, 2.3)
Metabolic factors												
High BMI	41 (20, 68)	253 (175, 337)	18.0 (20.4, 15.7)	3.9 (1.9, 6.6)	10.7 (7.2, 14.5)	6.0 (5.4, 6.6)	2045 (1055, 3369)	13,035 (9058, 17,287)	18.5 (20.8, 16.3)	171.2 (87.5, 284.8)	495.1 (340.2, 660.3)	6.5 (6.1, 6.9)
Environmental factors												
Ambient particulate matter pollution	12 (7, 20)	88 (60, 119)	21.7 (23.8, 19.6)	1.4 (0.7, 2.3)	4.3 (2.9, 5.7)	7.3 (6.9, 7.6)	514 (282, 844)	3761 (2547, 5174)	21.8 (23.6, 19.9)	47.6 (26.4, 78.4)	154.0 (104.5, 210.8)	7.7 (7.3, 8.2)
Household air pollution from solid fuels	39 (25, 64)	60 (37, 87)	1.8 (0, 3.7)	4.5 (2.9, 7.4)	2.9 (1.8, 4.3)	−1.2 (−3.5, 1.1)	1565 (983, 2620)	2436 (1507, 3599)	1.9 (0, 3.8)	146.1 (92.1, 242.2)	100.3 (62.5, 148.0)	−1.1 (−3.4, 1.3)
Tobacco												
Smoking	19 (15, 24)	50 (39, 60)	5.4 (4.5, 6.3)	2.0 (1.6, 2.5)	2.2 (1.7, 2.7)	0.3 (−0.7, 1.4)	836 (649, 1038)	2230 (1720, 2772)	5.8 (4.8, 6.7)	75.1 (58.4, 93.3)	88.5 (68.5, 109.7)	0.6 (−0.5, 1.7)
Second-hand smoke	21 (8, 32)	62 (24, 94)	6.9 (4.5, 9.2)	2.3 (0.9, 3.6)	3.0 (1.1, 4.5)	0.9 (−1.9, 3.7)	843 (317, 1318)	2604 (962, 4048)	7.2 (4.7, 9.7)	77.9 (29.4, 121.2)	106.0 (39.5, 164.3)	1.2 (−1.6, 4.0)
Dietary												
Diet low in fruits	19 (13, 25)	54 (37, 74)	6.3 (4.8, 7.8)	2.2 (1.5, 2.8)	2.6 (1.8, 3.6)	0.8 (−0.7, 2.3)	800 (550, 1072)	2317 (1552, 3207)	6.5 (5.0, 8.1)	73.6 (50.9, 98.7)	95.0 (63.9, 131.2)	1.0 (−0.6, 2.6)
Diet low in whole grains	10 (3, 14)	30 (10, 46)	7.6 (5.1, 10.0)	1.1 (0.4, 1.6)	1.5 (0.5, 2.2)	1.2 (−1.7, 4.0)	394 (137, 602)	1286 (450, 1992)	7.8 (5.3, 10.3)	36.9 (13.1, 56.2)	52.6 (18.2, 81.0)	1.5 (−1.4, 4.3)
Diet high in red meat	4 (1, 7)	15 (5, 24)	9.5 (7.3, 11.8)	0.5 (0.1, 0.7)	0.7 (0.2, 1.1)	1.9 (−1.1, 4.9)	171 (54, 282)	648 (215, 1050)	9.6 (7.1, 12.0)	15.8 (4.9, 26.0)	26.0 (8.6, 42.1)	2.2 (−0.7, 5.1)
Diet high in processed meat	5 (3, 6)	19 (13, 24)	9.7 (8.6, 10.8)	0.6 (0.4, 0.7)	0.9 (0.6, 1.1)	2.0 (0.7, 3.3)	214 (141, 273)	827 (551, 1068)	9.9 (8.6, 11.1)	19.7 (12.9, 25.3)	33.2 (22.1, 42.9)	2.4 (1.0, 3.8)
Diet high in sugar-sweetened beverages	5 (4, 7)	17 (11, 22)	7.0 (5.1, 9.0)	0.6 (0.5, 0.8)	0.8 (0.5, 1.0)	0.8 (−0.6, 2.2)	210 (153, 267)	694 (424, 957)	8.0 (5.6, 10.3)	19.8 (14.8, 25.0)	28.2 (17.5, 38.5)	1.5 (−0.2, 3.1)
Diet low in fibre	8 (5, 12)	21 (10, 33)	5.2 (2.7, 7.7)	1.0 (0.5, 1.4)	1.0 (0.5, 1.6)	0.2 (−2.2, 2.6)	331 (168, 492)	846 (384, 1323)	5.4 (2.9, 7.9)	30.9 (15.9, 45.7)	35.0 (16.0, 54.6)	0.4 (−2.0, 2.9)
Diet low in nuts and seeds	5 (1, 9)	15 (6, 26)	7.2 (5.2, 9.3)	0.5 (0.2, 1.1)	0.7 (0.3, 1.3)	1.1 (−2.0, 4.3)	207 (63, 394)	678 (256, 1208)	7.8 (5.7, 9.9)	19.1 (5.6, 36.7)	27.6 (10.5, 49.2)	1.5 (−1.5, 4.5)
Physical activity												
Low physical activity	14 (6, 24)	47 (23, 80)	8.5 (6.5, 0.5)	1.8 (0.9, 3.1)	2.5 (1.3, 4.2)	1.5 (−1.0, 4.0)	452 (201, 845)	1501 (694, 2690)	8.0 (5.8, 10.2)	47.4 (22.1, 86.6)	68.1 (32.7, 119.9)	1.5 (−1.1, 4.1)
UMICs												
All factors	195 (185, 207)	492 (452, 529)	5.3 (4.9, 5.7)	14.5 (13.6, 15.4)	15.5 (14.2, 16.7)	0.2 (−0.1, 0.6)	9244 (7760, 10,940)	23,228 (19,229, 28,120)	5.2 (4.4, 6.1)	589.0 (496.8, 696.7)	684.4 (566.8, 825.6)	0.6 (−0.3, 1.4)
Metabolic factors												
High BMI	73 (47, 102)	227 (159, 300)	7.3 (6.4, 8.1)	4.9 (3.1, 7.1)	6.8 (4.7, 9.1)	1.3 (−0.2, 2.9)	3712 (2309, 5457)	12,654 (8594, 17,371)	8.3 (7.4, 9.2)	225.3 (138.5, 331.6)	366 (249.6, 502.2)	2.2 (0.7, 3.6)
Environmental factors												
Ambient particulate matter pollution	20 (13, 29)	79 (56, 102)	10.1 (9.7, 10.5)	1.5 (1.0, 2.1)	2.5 (1.8, 3.2)	2.2 (1.0, 3.5)	915 (560, 1346)	3814 (2623, 5204)	10.9 (10.5, 11.4)	58.9 (36.4, 86.0)	111.9 (77.0, 152.7)	3.1 (1.8, 4.4)
Household air pollution from solid fuels	22 (14, 32)	16 (8, 27)	−0.9 (−3.1, 1.3)	1.6 (1.0, 2.3)	0.5 (0.3, 0.8)	−2.4 (−4.7, −0.1)	1081 (678, 1602)	737 (362, 1266)	−1.1 (−3.5, 1.3)	68.8 (43.4, 101.6)	21.6 (10.6, 37.0)	−2.4 (−4.8, 0.1)
Tobacco												
Smoking	24 (20, 28)	45 (36, 55)	3.1 (2.2, 4.1)	1.6 (1.4, 1.9)	1.4 (1.1, 1.6)	−0.6 (−1.5, 0.3)	1274 (1004, 1587)	2619 (1967, 3304)	3.6 (2.5, 4.8)	78.5 (61.8, 97.4)	75.1 (56.4, 94.6)	−0.1 (−1.3, 1.0)
Second-hand smoke	22 (9, 33)	44 (18, 68)	3.5 (1.0, 6.0)	1.6 (0.6, 2.5)	1.4 (0.5, 2.1)	−0.5 (−3.5, 2.5)	1044 (398, 1637)	2222 (826, 3547)	3.9 (1.2, 6.5)	66.5 (25.4, 103.9)	64.8 (24.1, 103.6)	−0.1 (−3.1, 2.9)
Dietary												
Diet low in fruits	11 (6, 16)	18 (8, 31)	2.3 (−0.5, 5.1)	0.8 (0.4, 1.2)	0.6 (0.2, 1.0)	−1.0 (−3.5, 1.6)	557 (311, 859)	842 (364, 1523)	1.8 (−1.0, 4.6)	35.2 (19.6, 54.2)	24.9 (10.8, 45.0)	−1.0 (−3.7, 1.7)
Diet low in whole grains	10 (4, 15)	25 (9, 37)	4.9 (2.3, 7.5)	0.8 (0.3, 1.1)	0.8 (0.3, 1.2)	0.1 (−2.9, 3.0)	480 (170, 748)	1190 (412, 1887)	5.1 (2.4, 7.8)	30.8 (11.0, 47.8)	34.9 (12.1, 55.4)	0.5 (−2.5, 3.5)
Diet high in red meat	13 (6, 18)	40 (24, 56)	7.4 (6.5, 8.4)	0.9 (0.5, 1.3)	1.3 (0.7, 1.8)	1.2 (−0.7, 3.1)	638 (333, 950)	2081 (1276, 2958)	7.8 (6.8, 8.8)	40.2 (20.9, 60.1)	60.7 (37.1, 86.3)	1.8 (−0.1, 3.6)
Diet high in processed meat	7 (4, 8)	20 (12, 24)	6.5 (5.0, 8.1)	0.5 (0.3, 0.6)	0.6 (0.4, 0.8)	0.8 (−0.8, 2.4)	347 (215, 461)	1033 (597, 1389)	6.8 (5.0, 8.6)	22.0 (13.7, 29.2)	30.1 (17.4, 40.6)	1.3 (−0.5, 3.1)
Diet high in sugar-sweetened beverages	7 (5, 9)	18 (11, 24)	5.4 (3.5, 7.4)	0.5 (0.4, 0.7)	0.6 (0.3, 0.8)	0.3 (−1.3, 2.0)	340 (235, 449)	872 (512, 1221)	5.4 (3.3, 7.5)	21.6 (14.9, 28.5)	25.7 (15.1, 35.9)	0.6 (−1.1, 2.4)
Diet low in fibre	5 (2, 9)	10 (4, 17)	3.1 (0.4, 5.8)	0.4 (0.2, 0.7)	0.3 (0.1, 0.5)	−0.7 (−3.7, 2.3)	274 (115, 442)	497 (190, 833)	2.8 (0, 5.6)	17.5 (7.3, 28.1)	14.7 (5.6, 24.7)	−0.5 (−3.6, 2.5)
Diet low in nuts and seeds	4 (1, 8)	9 (2, 18)	3.7 (0.2, 7.3)	0.3 (0.1, 0.6)	0.3 (0.1, 0.6)	−0.4 (−4.6, 3.7)	200 (34, 423)	397 (66, 844)	3.4 (−0.3, 7.0)	12.9 (2.2, 27.2)	11.7 (2.0, 24.9)	−0.3 (−4.5, 3.9)
Physical activity												
Low physical activity	17 (9, 28)	47 (24, 75)	6.2 (4.2, 8.2)	1.4 (0.7, 2.2)	1.5 (0.8, 2.5)	0.4 (−2.0, 2.9)	638 (303, 1102)	1749 (870, 3010)	6.0 (3.9, 8.2)	43.7 (21.4, 75.0)	52.7 (26.3, 89.9)	0.7 (−1.9, 3.3)
HICs												
All factors	165 (155, 171)	242 (217, 256)	1.6 (1.2, 2.0)	12.7 (11.9, 13.2)	9.7 (8.8, 10.2)	−0.8 (−1.1, −0.5)	6407 (5343, 7626)	12,403 (9723, 15,525)	3.2 (2.1, 4.3)	508.3 (422.6, 607.0)	601.7 (467.5, 753.6)	0.6 (−0.4, 1.7)
Metabolic factors												
High BMI	68 (44, 93)	109 (75, 147)	2.1 (0.6, 3.5)	5.3 (3.4, 7.2)	4.7 (3.4, 6.2)	−0.4 (−2.0, 1.3)	3237 (2146, 4489)	7260 (5018, 10,010)	4.3 (3.0, 5.6)	262.2 (174.3, 361.8)	375.3 (258.2, 516.2)	1.5 (0, 3.0)
Environmental factors												
Ambient particulate matter pollution	23 (13, 33)	26 (16, 37)	0.4 (−1.5, 2.3)	1.7 (1.0, 2.5)	1.0 (0.7, 1.5)	−1.4 (−3.7, 0.8)	868 (504, 1290)	1291 (762, 1996)	1.7 (−0.3, 3.7)	68.8 (39.9, 102.5)	63.0 (36.8, 97.5)	−0.3 (−2.5, 1.9)
Household air pollution from solid fuels	2 (1, 3)	0 (0, 1)	−2.5 (−5.7, 0.7)	0.1 (0.1, 0.2)	0 (0, 0)	−2.9 (−6.3, 0.4)	62 (32, 107)	21 (6, 50)	−2.3 (−5.5, 1.0)	5.0 (2.5, 8.6)	1.0 (0.3, 2.4)	−2.7 (−6.1, 0.6)
Tobacco												
Smoking	22 (18, 26)	21 (17, 27)	−0.1 (−1.1, 1.0)	1.7 (1.4, 2.0)	0.9 (0.8, 1.2)	−1.5 (−2.6, −0.4)	1067 (809, 1377)	1489 (1068, 1974)	1.4 (0, 2.7)	86.4 (65.4, 111.7)	76.2 (54.5, 101.1)	0 (−3.1, 3.1)
Second-hand smoke	10 (4, 16)	12 (4, 18)	0.6 (−2.3, 3.5)	0.8 (0.3, 1.2)	0.5 (0.2, 0.8)	−1.3 (−4.5, 2.0)	471 (172, 759)	741 (261, 1239)	2.0 (−0.9, 4.9)	38.3 (14.1, 61.9)	38.1 (13.5, 63.8)	−0.4 (−1.8, 1.0)
Dietary												
Diet low in fruits	8 (3, 12)	10 (4, 16)	0.9 (−1.9, 3.7)	0.6 (0.3, 0.9)	0.4 (0.2, 0.7)	−1.2 (−4.2, 1.9)	306 (143, 515)	519 (226, 940)	2.4 (−0.3, 5.1)	24.5 (11.5, 41.3)	25.7 (11.2, 46.4)	0.2 (−2.7, 3.0)
Diet low in whole grains	9 (3, 13)	13 (5, 19)	1.6 (−1.2, 4.4)	0.7 (0.2, 1.0)	0.5 (0.2, 0.8)	−0.8 (−4.0, 2.3)	336 (117, 524)	651 (209, 1056)	3.2 (0.3, 6.2)	26.6 (9.2, 41.6)	31.4 (10.1, 51.2)	0.6 (−2.5, 3.7)
Diet high in red meat	16 (11, 22)	24 (16, 32)	1.6 (0, 3.1)	1.3 (0.9, 1.7)	1.0 (0.6, 1.3)	−0.8 (−2.5, 0.9)	670 (446, 931)	1292 (825, 1864)	3.2 (1.5, 4.9)	53.4 (35.4, 74.1)	63.6 (40.5, 91.7)	0.7 (−1.1, 2.4)
Diet high in processed meat	20 (15, 25)	30 (21, 38)	1.6 (0.4, 2.9)	1.6 (1.1, 1.9)	1.2 (0.9, 1.5)	−0.8 (−2.2, 0.6)	822 (557, 1099)	1660 (1090, 2302)	3.5 (1.9, 5.1)	65.3 (44.2, 87.4)	80.8 (52.6, 112.9)	0.8 (−0.8, 2.5)
Diet high in sugar-sweetened beverages	6 (3, 9)	12 (6, 16)	2.8 (0.9, 4.6)	0.5 (0.3, 0.7)	0.5 (0.3, 0.7)	−0.1 (−2.3, 2.0)	270 (140, 386)	681 (358, 1002)	5.3 (3.3, 7.2)	21.7 (11.3, 30.9)	34.6 (18.5, 51.3)	2.1 (0, 4.1)
Diet low in fibre	6 (2, 9)	8 (3, 12)	1.2 (−1.6, 4.0)	0.4 (0.2, 0.7)	0.3 (0.1, 0.5)	−1.1 (−4.2, 2.1)	207 (79, 343)	364 (133, 620)	2.6 (−0.2, 5.4)	16.4 (6.3, 27.3)	17.5 (6.3, 29.9)	0.2 (−2.9, 3.3)
Diet low in nuts and seeds	3 (1, 7)	4 (1, 8)	0.7 (−3.1, 4.6)	0.2 (0.1, 0.5)	0.1 (0, 0.3)	−1.4 (−5.8, 3.0)	116 (24, 242)	189 (37, 396)	2.1 (−1.5, 5.8)	9.2 (1.9, 19.1)	9.0 (1.7, 18.9)	−0.1 (−4.1, 4.0)
Physical activity												
Low physical activity	18 (9, 29)	27 (14, 44)	1.9 (−0.5, 4.2)	1.4 (0.7, 2.3)	1.0 (0.5, 1.7)	−0.8 (−3.6, 1.9)	571 (258, 1025)	1146 (539, 2037)	3.5 (1.0, 5.9)	44.7 (20.1, 81.4)	53.6 (24.8, 98.2)	0.7 (−2.1, 3.5)

### Global type 2 diabetes by geographical regions

**Fig. 1:**
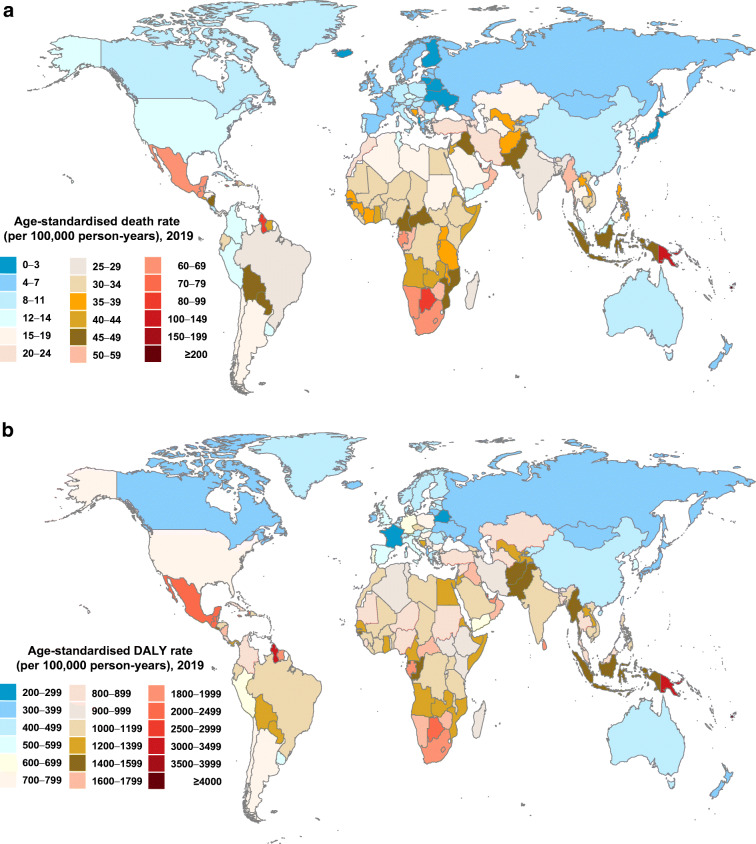
Age-standardised death (**a**) and DALY (**b**) rate of type 2 diabetes per 100,000 person-years by country and territory, 2019

Figure 1 demonstrates significant geographical variations of the type 2 diabetes burden worldwide. In 2019, the country with the highest age-standardised death rate (ASDR) and age-standardised DALY rates was Fiji (257.4 [95% CI 210.3, 309.2]/100,000 person-years and 6884.3 [5667.8, 8214.8]/100,000 person-years, respectively), whereas the country with the lowest ASDR was Japan (2.0 [1.7, 2.1]/100,000 person-years) and the lowest age-standardised DALY rates was France (278.2 [220.6, 345.7]/100,000 person-years). When stratified by the World Bank regions, ASDR in the Oceania region was the highest (121.0/100,000 person-years), and in high-income Asia Pacific countries (Japan, South Korea, Singapore and Brunei) was the lowest (4.2/100,000 person-years). Age-standardised DALY rates were highest in the Oceania region (3703.4/100,000 person-years) and lowest in the Eastern Europe region (376.0/100,000 person-years).

### Global type 2 diabetes by country incomes

In 2019, type 2 diabetes-related ASDR in low- and lower-middle-income countries (LICs: 33.0 [95% CI 29.6, 36.9]/100,000 person-years and LMICs: 31.2 [28.5, 33.9]/100,000 person-years) was 2–3 times higher than those in upper-middle- and high-income countries (UMICs: 15.5 [14.2, 16.7]/100,000 person-years; HICs: 9.7 [8.8, 10.2]/100,000 person-years). Similarly, in 2019, the highest type 2 diabetes-related age-standardised DALY rate was reported in LICs (1050.9 [895.7, 1238.9]/100,000 person-years) and LMICs (1108.3 [951.6, 1285.5]/100,000 person-years), which were nearly double the burdens in UMICs (684.4 [566.8, 825.6]/100,000 person-years) and HICs (601.7 [467.5, 753.6]/100,000 person-years, Table 1).

**Fig. 2:**
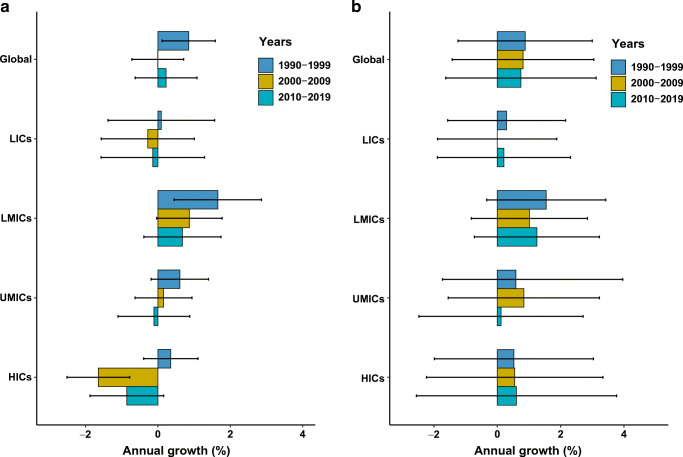
Annualised rate of change in ASDR (**a**) and age-standardised DALY rate (**b**) of type 2 diabetes, globally, and by different income countries, 1990–1999, 2000–2009 and 2010–2019

Globally, the annual growth for ASDR and age-standardised DALY rates of type 2 diabetes remained stable over time (0.9% [95% CI 0.1, 1.6] and 0.9% [−1.2, 3.0] in 1990–1999, 0.0% [−0.7, 0.7] and 0.8% [−1.4, 3.0] in 2000–2009, 0.2% [−0.6, 1.1] and 0.7% [−1.6, 3.1] in 2010–2019, respectively) (*p*_trend_ >0.05) (Fig. 2a,b). The annual growth rates for ASDR and age-standardised DALY rate showed no significant differences across all three time periods in all income tiers (Fig. 2a,b).

### PAFs for type 2 diabetes

**Fig. 3:**
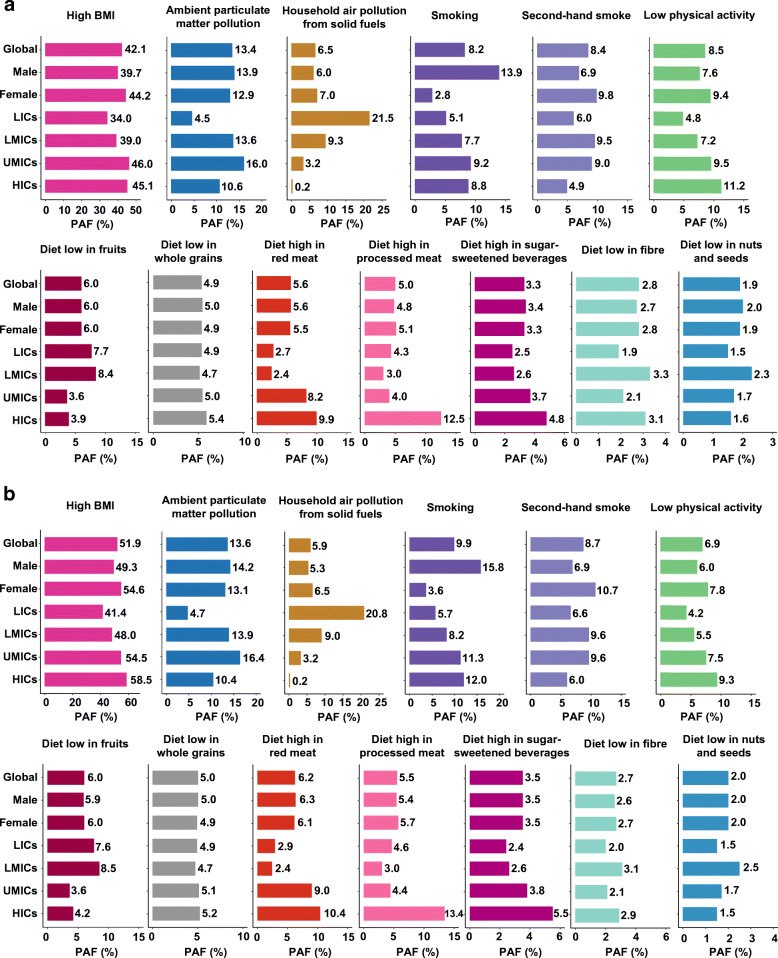
PAFs of death (**a**) and DALYs (**b**) attributed to risk factors for type 2 diabetes, 2019

Globally, the leading risk factor for type 2 diabetes-related ASDR was high BMI. The ASDR attributable to high BMI had increased from 5.0 (95% CI 3.0, 7.3)/100,000 person-years to 7.6 (5.3, 10.0)/100,000 person-years during 1990–2019. This was followed by ambient particulate matter pollution (increased from 1.6 [1.0, 2.2] to 2.5 [1.7, 3.2]/100,000 person-years) and low physical activity (increased from 1.5 [0.8, 2.5] to 1.6 [0.8, 2.7]/100,000 person-years) (Table 1). The three risk factors collectively contributed to over half (54.1%) of all type 2 diabetes-related ASDR in 2019 globally (Fig. 3a). Similarly, age-standardised DALY rates attributable to high BMI increased from 224.8 (137.9, 332.0)/100,000 person-years to 411.1 (287.5, 552.2)/100,000 person-years during 1990–2019, followed by ambient particulate matter pollution (increased from 58.4 [36.6, 83.1] to 109 [74.1, 147.2] /100,000 person-years) and smoking (reduced from 79.2 [61.9, 97.9] to 78.1 [59.6, 98.7]/100,000 person-years) (Table 1). The three factors collectively contributed to 62.6% of type 2 diabetes-related DALYs globally in 2019 (Fig. 3b). With the exception of smoking and second-hand smoke, the proportions of deaths and DALYs attributable to type 2 diabetes varied less by sex globally in 2019 (Fig. 3a, b).

In LICs, the leading risk factor for type 2 diabetes-related ASDR was high BMI (ASDR attributable to high BMI increased from 6.2 [95% CI 2.7, 10.9]/100,000 person-years to 9.6 [5.7, 14.2]/100,000 person-years during 1990–2019). This was followed by household air pollution from solid fuels (reduced from 9.4 [5.7, 18.5] to 7.1 [4.7, 11.2]/100,000 person-years) and low fruits in the diet (stabilised at 2.6/100,000 person-years during 1990–2019) (Table 1, Fig. 3a). The leading risk factors contributing to age-standardised DALY rates were similar (Table 1, Fig. 3b).

In LMICs, high BMI remained the leading risk factor for type 2 diabetes-related deaths (ASDR attributable to high BMI increased from 3.9 [95% CI 1.9, 6.6] to 10.7 [7.2, 14.5]/100,000 person-years during 1990–2019). This was followed by ambient particulate matter pollution (increased from 1.4 [0.7, 2.3] to 4.3 [2.9, 5.7]/100,000 person-years) and second-hand smoke (increased from 2.3 [0.9, 3.6] to 3.0 [1.1, 4.5]/100,000 person-years) (Table 1, Fig. 3a). The leading risk factors contributing to age-standardised DALY rates were similar (Table 1, Fig. 3b).

In UMICs, high BMI remained the leading risk factor attributable to type 2 diabetes-related deaths (ASDR attributable to high BMI increased from 4.9 [95% CI 3.1, 7.1] to 6.8 [4.7, 9.1]/100,000 person-years during 1990–2019). This was followed by ambient particulate matter pollution (increased from 1.5 [1.0, 2.1] to 2.5 [1.8, 3.2]/100,000 person-years) and low physical activity (increased from 1.4 [0.7, 2.2] to 1.5 [0.8, 2.5]/100,000 person-years) (Table 1, Fig. 3a) However, we observed a slightly different contribution pattern in age-standardised DALY rates, with high BMI, ambient particulate matter pollution and smoking being the leading risk factors (Table 1, Fig. 3b).

In HICs, despite high BMI remaining the leading risk factor for type 2 diabetes-related ASDR, its contribution was substantially lower than other income tiers and decreasing over time (ASDR attributable to high BMI reduced from 5.3 [95% CI 3.4, 7.2]/100,000 person-years to 4.7 [3.4, 6.2]/100,000 person-years during 1990–2019). This was followed by diet high in processed meat (reduced from 1.6 [1.1, 1.9] to 1.2 [0.9, 1.5]/100,000 person-years) and low physical activity (reduced from 1.4 [0.7, 2.3] to 1.0 [0.5, 1.7]/100,000 person-years) (Table 1, Fig. 3a). For age-standardised DALY rates, high BMI, diet high in processed meat and smoking are leading risk factors, and all three factors demonstrated flattening the upward tendency over time (Table 1, Fig. 3b).

Our study analysed the correlation between per capita gross domestic product (GDP) and the attributable burden of risk factors in 203 countries in 2019 (electronic supplementary material [ESM] Table 1). Per capita GDP was positively correlated with PAFs for death and DALYs caused by diet high in red meat, diet high in processed meat, diet high in sugar-sweetened beverages and low physical activity (all *p*<0.0001), but negatively correlated with PAFs for death and DALYs caused by household air pollution from solid fuels, smoking and diet low in fruits (all *p*<0.0001). However, per capita GDP showed a negative correlation with PAFs only for DALYs caused by second-hand smoke (*p*<0.0001).

## Discussion

Our study showed that type 2 diabetes-related mortality and DALYs varied substantially during 1990–2019, globally and by income tiers. The numbers of deaths and DALYs attributable to type 2 diabetes doubled from its 1990 levels to 1.47 million and 66.3 million in 2019. ASDR and age-standardised DALY rate also increased by 10.8% and 27.6% to 18.5/100,000 and 801.5/100,000, respectively, during the same period. Overall, type 2 diabetes-related ASDR and age-standardised DALY rate increased with the declining income tier of the countries. Both rates in LICs and LMICs were 2–3 times higher than those in UMICs and HICs in 2019. Further, LMICs reported the largest increase in the average annual growth of ASDR (1.3%) and an age-standardised DALY rate (1.6%) of type 2 diabetes. In contrast, the annual growth of ASDR and age-standardised DALY rate was the lowest (−0.8% and nearly zero, respectively) in HICs, during 1990–2019. BMI is the key common leading risk factor of type 2 diabetes disease burden across all income tiers. With the exception of BMI, while in low- and middle- (lower-middle- and upper-middle-) income countries, risk factors attributable to type 2 diabetes-related deaths and DALYs are mostly environment-related, the risk factors in HICs are mostly lifestyle-related.

Our study demonstrated a rapidly growing trend of type 2 diabetes burden in low- and middle-income country settings, although concerns related to type 2 diabetes burden have historically focused on HICs, which was consistent with previous studies [29, 30]. Both ASDR and age-standardised DALY rate in LICs and LMICs are consistently 2–3 times higher than those in UMICs and HICs throughout our study duration. In particular, the fact that HICs reported a negative or nearly zero annual growth in both age-standardised rates whereas LMICs have more significant positive annual growth suggests that the gap in disease burden across income tiers will likely widen further in the future. This poses significant public health challenges to LICs and LMICs. First, compared with higher income countries, LICs and LMICs have limited medical resources and poor access to therapeutic drugs for type 2 diabetes prevention and treatment. Allocation of health resources for type 2 diabetes control and prevention in these countries are often insufficient. Second, in LICs and LMICs, the poor health awareness of patients with diabetes may result in significant delays in diagnosis and treatment of diabetes, leading to a severe burden of diabetes disability and related complications. Third, PAFs for death and DALYs differed by income tiers, as indicated by our study. Exposure to environmental risk factors for diabetes is more common in lower income countries as a result of limited options of energy consumption in these countries. If the current trend continues, type 2 diabetes will likely cause an increasingly severe disease burden in lower income countries than their higher income counterparts.

Other studies have also demonstrated similar associations between income tiers and type 2 diabetes-related mortality and DALYs. Lin et al [31] reported that, worldwide, the rates of mortality and disability caused by type 2 diabetes exhibited an upward trend in low-income and lower-middle-income countries. In comparison, the mortality rate due to type 2 diabetes in HICs, such as Australia [11] and Sweden [10], have shown a declining trend. Consistently, a separate study reported a large reduction in complications related to type 2 diabetes in HICs during 2000–2015, indicating an alleviation of disability caused by type 2 diabetes in these countries [32]. Lin et al [31] reported that for type 2 diabetes, the association between mortality or DALY rates with Sociodemographic Index (SDI) demonstrated an inverse U-shaped curve with the higher rates occurring in low-middle, middle, and high-middle SDI countries. The corresponding rates in low-SDI and high-SDI countries were substantially lower.

Our study indicates that BMI remains the leading risk factor for type 2 diabetes-related deaths and DALYs globally and across all income tiers. Obesity is a well-documented important public health issue and a key contributor to numerous chronic diseases [33, 34]. Historically, the increase in the prevalence of obesity began in HICs in the 1970s, followed by most middle-income countries, and more recently, low-income countries [35]. The increase in obesity prevalence is likely a consequence of economic development and affects the occurrence of type 2 diabetes. However, economic development and wealth can enable better type 2 diabetes intervention. Over the past decade, lifestyle interventions in HICs, aiming to reduce the risk of overweight and obesity, have reportedly reduced the risk of type 2 diabetes, as demonstrated by the US Diabetes Prevention Program [36] and the Dutch Diabetes Prevention Study [37]. The American Cancer Society’s Cancer Prevention Study also reported that intentional weight loss was associated with a 28% reduction in type 2 diabetes-related mortality and its complications (cardiovascular disease) [38]. In contrast, prevention programmes for overweight and obesity in low- and middle-income countries have commenced later than high-income countries. In 2016, the Chinese government declared the ‘Healthy China 2030’ initiative, aiming to facilitate appropriate diet and physical activities to reduce obesity and hence type 2 diabetes in the Chinese population. A steady growing trend of new type 2 diabetes has been documented during 1990–2017 [39], which is closely related to the rise in prevalence of overweight and obesity in China in recent years [40]. The country with the largest number of adults with diabetes aged 20–79 years in 2021 is China, and it accounts for 26.2% of the number of people living with diabetes globally [41].

Our study shows that both household and ambient air pollution are major risk factors for type 2 diabetes deaths and DALYs in LICs and LMICs, consistent with previous findings [42]. Pollution and poverty are closely related. An estimated 3 billion people in low- and middle-income countries, mostly in rural communities, still use solid fuels (firewood, biomass or charcoal) and traditional stoves for heating and cooking [43]. This results in large populations being exposed to household air pollution and subsequent type 2 diabetes onset and complications because air pollution is a leading cause of insulin resistance [44]. Ambient particulate matter pollution, often measured by the air density of particulate matter 2.5 with an aerodynamic diameter less than 2.5 μm (PM_2.5_), has led to 4.2 million deaths and 103.1 million DALYs in 2015 [45]. Ambient particulate matter pollution disproportionately affects the poor and the vulnerable in low-income and middle-income countries, which account for 79% of adults living with diabetes [1, 42]. The population is often neglected in these settings where economic development is prioritised, and its health impacts on people living with diabetes are largely underestimated. Our study demonstrates significant contributions of both household and ambient air pollution to type 2 diabetes and promotes sustainable green development and pollutant reduction.

Our study also demonstrates that diet rich in processed meat is the second most important risk factor for type 2 diabetes-related deaths and DALYs in HICs. Meta-analysis studies have consistently shown that diet rich in processed meat is associated with an increased risk of type 2 diabetes in HICs [46, 47]. Over the past decade, despite some HICs having witnessed declining trends in the consumption of red and processed meat, consumption in these countries remains higher than in other income countries [48]. McMichael et al found that the global average consumption was 100 g per person per day, whereas the consumption now is as high as 200–250 g in HICs [49]. However, as global meat prices have fallen, red and processed meat has become increasingly available in low- and middle-income countries [48]. At the same time, health-motivated tax on red and processed meat is low in these countries [50]. This could potentially increase consumption of processed meat and hence the disease burden of type 2 diabetes in low- and middle-income countries.

Our study has several limitations. First, the projection relied heavily on estimates driven by GBD 2019; therefore, similar limitations in estimates of deaths, DALYs and attributable burden in the GBD study also apply to this study. Second, it deviates somewhat from the comparative risk assessment approach when the GBD study estimates the burden for total particulate matter pollution and divides this total burden proportionately between the ambient and household particulates. Third, for exposure measurement, patterns of data availability are non-uniform across geography and over time. Although the GBD study has modified this, it still causes a bias in comparison.

### Conclusions

Type 2 diabetes disease burden has increased globally in countries of all income tiers. High BMI, ambient particulate matter pollution and low physical activity collectively contributed to half of type 2 diabetes-related deaths and two-thirds of type 2 diabetes-related DALYs globally in 2019. BMI remains the most important contributing factor to the type 2 diabetes disease burden. Following BMI, the environmental factors are the next important contributor to the type 2 diabetes burden in low- and lower-middle-income countries, whereas dietary factors are the next important contributing factor in HICs.

## Supplementary information

ESM 1 (PDF 158 kb)

## Data Availability

To download the data used in these analyses, please visit the Global Health Data Exchange at http://ghdx.healthdata.org/gbd-2019 [51].

## Abbreviations

ASDR: Age-standardised death rate
DALYs: Disability-adjusted life years
GBD: Global Burden of Disease
GDP: Gross domestic product
HICs: High-income countries
LICs: Low-income countries
LMICs: Lower-middle-income countries
PAF: Population attributable fraction
SDI: Sociodemographic Index
UMICs: Upper-middle-income countries
YLDs: Years lived with disability
YLLs: Years of life lost
