# Supplementary material for: Low- and middle-income countries demonstrate rapid growth of type 2 diabetes: an analysis based on Global Burden of Disease 1990–2019 data
Source: Diabetologia. 2022 May 19;65(8):1339–52. doi: 10.1007/s00125-022-05713-6 (PMC9118183; doi:10.1007/s00125-022-05713-6)
Supplement: Supplementary file 1 — (PDF 158 kb) [file 125_2022_5713_MOESM1_ESM.pdf]

Table 1. The Pearson correlation between per capita GDP and attributable burden of risk factors in 203 countries in 2019

Variable	PAF	r	*P*
**Death**			
Metabolic factors	High BMI	-0.060	0.403
Environmental factors	Ambient particulate matter pollution	-0.042	0.564
	Household air pollution from solid fuels	-0.472	<0.0001
Tobacco	Smoking	-0.358	<0.0001
	Second-hand smoke	0.116	0.108
Dietary	Diet low in fruits	-0.296	<0.0001
	Diet low in whole grains	-0.042	0.562
	Diet high in red meat	0.577	<0.0001
	Diet high in processed meat	0.496	<0.0001
	Diet high in sugar-sweetened beverages	0.651	<0.0001
	Diet low in fiber	0.130	0.071
	Diet low in nuts and seeds	0.020	0.777
Physical activity	Low physical activity	0.454	<0.0001
**DALYs**			
Metabolic factors	High BMI	0.075	0.300
Environmental factors	Ambient particulate matter pollution	-0.039	0.588
	Household air pollution from solid fuels	-0.473	<0.0001
Tobacco	Smoking	-0.294	<0.0001
	Second-hand smoke	-0.277	<0.0001
Dietary	Diet low in fruits	-0.296	<0.0001
	Diet low in whole grains	-0.035	0.629
	Diet high in red meat	0.594	<0.0001
	Diet high in processed meat	0.504	<0.0001
	Diet high in sugar-sweetened beverages	0.669	<0.0001
	Diet low in fiber	0.107	0.137
	Diet low in nuts and seeds	-0.018	0.806
Physical activity	Low physical activity	0.398	<0.0001
